# An Easy Solution for Difficult Urethral Catheter: Three-Way Urethral Catheter Insertion Using a Guidewire Technique

**DOI:** 10.7759/cureus.32563

**Published:** 2022-12-15

**Authors:** Sima Patel, Satpal Antil

**Affiliations:** 1 Surgery, New Cross Hospital, Wolverhampton, GBR; 2 Urology, Walsall Manor Hospital, Walsall, GBR

**Keywords:** junior doctor, medical skills, catheter insertion, rubber catheter, urology

## Abstract

Urethral catheter insertion is a skill taught to all medical students but often not practised for a multitude of reasons. The difficult catheter can be a clinical nightmare for the junior doctor, especially on call, and can lead to significant mortality and morbidity with suboptimal repeated attempts.

The incorporation of a soft-tipped hydrophilic guidewire technique for the insertion of a two-way urethral catheter has been described in the literature and has the potential to reduce the morbidity and mortality of patients by making the insertion of a catheter less traumatic. Here, we propose and describe the insertion of a three-way urethral catheter, performed using the technique employed for the insertion of a two-way urinary catheter via the use of a hydrophilic guidewire, with similar outcomes. A hydrophilic soft-tipped guidewire to insert a three-way urethral catheter can be used in the wards, in the emergency department, and in a theatre setting. The district hospital in which this method was employed demonstrated a 100% success rate in the insertion of a urethral catheter (N = 15), with 26% of cases (four patients out of 15) having a three-way urethral catheter inserted using the soft-tipped hydrophilic guidewire method. Follow-ups of these patients showed that there were no complications or adverse effects experienced by the patients.

The use of a soft-tipped guidewire approach to insert a difficult urethral catheter can reduce the financial burden on the healthcare system by eliminating costs due to harm/trauma caused by repeated unsuccessful urethral catheter attempts or those attempts that have been performed suboptimally and have led to potential patient harm. The use of a hydrophilic guidewire-assisted technique to insert a three-way urinary catheter is a safe and easy option for those who have had repeated unsuccessful attempts. The hydrophilic guidewire approach has the potential to reduce morbidity and mortality associated with urethral catheterisation and improve patient safety.

## Introduction

The insertion of a urethral catheter is a basic skill taught to medical students in universities around the world. However, urethral catheterisation can often be more challenging than other core procedures taught at medical schools, such as cannulation and venepuncture. Some of the reasons for the increased challenges with this procedure include that the skill is utilised less often than other skills, there is a lack of direct visualisation of the urethral catheter and internal anatomy of the patient when the urethral catheter is inserted into the meatus, and there is often an element of embarrassment/awkwardness from the patient which may affect the clinician when inserting a urethral catheter [1]. These barriers to urethral catheter insertion contribute to a lack of confidence and a lack of experience associated with this skill, with individuals shying away from performing the procedure. In our experience, the reluctance to insert a urethral catheter is more commonly experienced when a three-way urethral catheter needs to be inserted. Part of this reluctance may be due to the above-mentioned reasons; however, as an additional factor, clinicians may also be intimidated by the size of the urethral catheter being inserted. This is because a three-way urethral catheter is traditionally larger than a two-way urethral catheter. Traditionally, three-way urethral catheters measure 18-F or above. An area for further work would be on the relationship between catheter size and confidence in performing three-way catheter insertion as in reality the size difference is clinically insignificant if the correct approach is adopted. The insertion of a urethral catheter is required for multiple reasons within the clinical practice, including decompression of the urinary bladder in cases of urinary retention, fluid monitoring in an unwell patient, perioperative or intraoperative monitoring of fluid balance, and in cases of frank haematuria which require a three-way catheter insertion for both monitoring and treatment.

In general, urethral catheterisation can be difficult in both male and female patients. In male patients, physiological reasons for difficulties during the process include the following; a buried penis, tight foreskin/phimosis, urethral strictures (possible along the entire length of the penis, including penile, bulbar, and membranous urethra), prostate urethra (which can be affected by conditions such as benign prostate hyperplasia, prostate cancer, and possible fibrosis and strictures as a result of injury due to multiple attempts at instrumentation of the urethra), and at the bladder neck. In female patients, difficulties encountered are mainly due to atrophy of the anterior urethra often seen in post-menopausal patients. This atrophy of the anterior urethra can lead to anatomy distortion. Female patients anatomically have a shorter urethra and are less likely to be affected by urethral strictures, although this may be a possible barrier to a simple urethral catheterisation. Urethral catheterisation difficulties, in both male and female patients, are usually referenced when discussing the insertion of a two-way urethral catheter. However, these issues can be exacerbated during the insertion of three-way urethral catheters due to the increase in the size of the urethral catheter and/or lack of confidence or experience in the procedure. Urethral catheterisation has the potential to cause significant morbidity and mortality to the patient. Potential adverse effects of repeated urethral catheterisation attempts, or those performed suboptimally, include bacterial colonisation, chronic infection and urethral damage (including false passages), damage to the prostate urethra, pain and discomfort experienced by the patient, and possible permanent urological damage such as urethral strictures [2-5].

Increased frequency of urethral catheterisation increases the risk due to repeat procedures/attempts by inexperienced hands [6]. With each attempt, a new set of instruments/urethral catheter is required to be in keeping with the aseptic, non-touch method employed within the National Health Service (NHS), to minimise the risk of infection [7].

Finding a solution to minimise attempts at urethral catheterisation would not only improve patient satisfaction but will also minimise the potential risk of infection and harm to the patient. A solution would also be incredibly cost-effective when compared to prolonged hospital admissions and/or the need for future intervention in those who have been impacted due to suboptimal techniques and/or multiple unsuccessful attempts.

It is worth mentioning that the technique described in this study should be employed with clinical caution. Any concerns of urethral injury would make the below technique inappropriate to employ. Features consistent with urethral injury include blood at the urethral meatus, high-riding prostate on digital rectal examination, scrotal or perineal ecchymosis, and/or mechanism of injury suggestive of a urethral history. Any concerns of urethral injury should prompt the use of a retrograde urethrogram to further investigate the potential of an injury.

## Technical report

The materials required for soft-tipped hydrophilic guidewire-assisted insertion of a three-way urethral catheter are shown in Table 1.

**Table 1:** Materials required for the hydrophilic guidewire-assisted technique of catheterisation. *: Catheter packs usually contain one sterile gauze, six sterile cotton balls, two plastic pots, one sterile sheet, and a sterile tray. **: The use of the word gauge, when used in reference to cannulas, is used to describe the size of the hole (inner diameter) of the needle. As the gauge number increases, the needle calibre decreases. ***: Pure soft-tipped hydrophilic guidewire, as well as a hybrid soft-tipped hydrophilic guidewire, can be utilised in the described technique. ****: A urinary catheter circumference size is measured in cm with the size of the catheter given in French. Each French is equivalent to 0.33 m [5].

Items required for hydrophilic guidewire technique	Quantity required
Catheter pack*	One
Sterile lidocaine gel	One
18 Gauge (18 G)**, Green cannula	One
Soft-tipped hydrophilic guidewire*	One
Urethral dilator 10–16 F/16 F	One
Catheter including three-way balloon retention catheter – 18 F**** Please note an 18-F three-way balloon retention catheter is used in the images below for demonstration	One
Urine bag	One

The hydrophilic soft-tipped hydrophilic guidewire used and recommended by the authors is the one used in the urology department, intraoperatively, for the insertion of ureteric stents.

**Figure 1:**
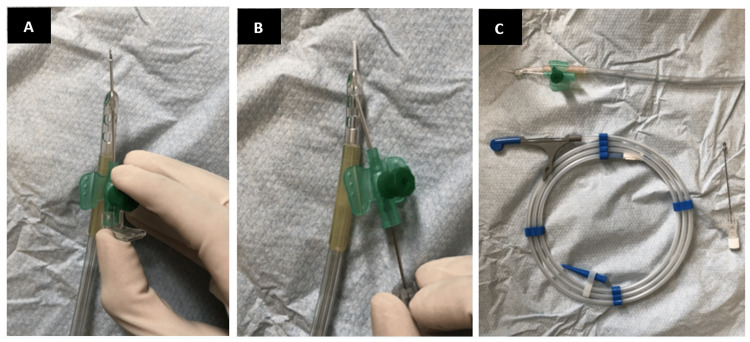
(A) The tip of the urethral catheter is pierced with a cannula*. (B) The cannula plug is removed and disposed of in the sharps bin. (C) The plastic tubing of the cannula is left in the urethral catheter. The three-way urethral catheter is now ready for use. Figure 1C shows the finished product of the above steps and the soft-tipped hydrophilic guidewire required for the next steps. *: 18-French three-way catheter has been used for demonstration.

**Figure 2:**
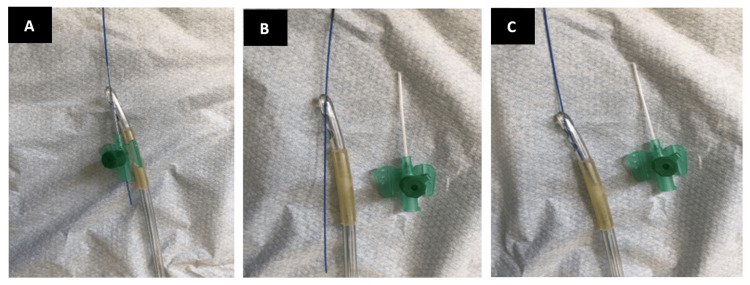
(A) Feed the distal, the non-soft-tipped, end of the guidewire through the plastic tip of the cannula. (B) Remove the cannula leaving the hydrophilic guidewire tip in the lumen of the catheter. (C) Feed the hydrophilic guidewire up through the catheter.

1. Prepare the urethral opening as you would for any urethral catheterisation attempt. This involves using a non-touch, aseptic technique to clean the urethral opening and applying the sterile sheet over the prepared area to create a sterile field.

2. Insert the sterile lidocaine gel into the urethral opening and wait for the appropriate time for the anaesthetic to take effect.

3. Prepare the urethral catheter. Pierce the tip of the urethral catheter with an 18-G cannula, adhering to sharp safety at all times and removing the sharp, needle part of the cannula and disposing of it appropriately while leaving the plastic tubing in situ (Figure 1).

4. Insert the soft, proximal end of the soft-tipped hydrophilic guidewire into the urethral opening and into the urinary bladder. In men, no more than 30 cm should be inserted to prevent coiling in the bladder [7]. In our experience and practice, it has been observed that if the distal end of the soft-tipped hydrophilic guidewire is at level with the end of the patient’s feet (approximately), the wire has passed the prostate and is likely in the bladder [8].

5. Railroad the urethral dilators over the hydrophilic guidewire, starting with 6-10-F and then 10-16-F. Correct placement will be noted when urine is seen at the distal end of the urethral dilator when held in position, which may take between three and five minutes. Step five is not deemed a necessary step and can be omitted. We have included it because this technique was used in the cases we performed (n = 15). Patient discomfort is a limiting factor in this step, and if experienced or encountered, urethral dilator insertion should be stopped and abandoned, and the clinician should move on to step six. Is it important to note that the correct dilation technique requires dilatation to be performed up to 2-F larger than the catheter to be placed; however, we were limited due to resource availability.

6. Carefully remove the urethral dilator while stabilising the hydrophilic guidewire to not displace its position in the bladder and feed the distal tip of the hydrophilic guidewire into the plastic tubing of the cannula, as shown in Figure 2A. Once 2-3 cm of the distal, firm tip of the hydrophilic soft-tipped guidewire (the distal end being usually firm) has been fed through the tip catheter via the cannula, remove the cannula from the tip of the catheter, leaving the hydrophilic guidewire in the catheter lumen, and feed the hydrophilic guidewire through the rest of the catheter (Figures 2B, 2C).

It is important to note that difficulty can be experienced in passing the guidewire through the cannula tip. There are other methods to overcome this difficulty such as cutting the tip of the catheter. This is out of the scope of this article but is important to bear in mind.

7. Railroad the catheter over the hydrophilic guidewire into the bladder. Water for injection or normal saline can be used to help lubricate the soft-tipped hydrophilic guidewire for ease of passage through the urethral catheter if resistance is felt. Full lubrication of the urethral catheter via the use of sterile lidocaine gel can aid in advancing the catheter into the bladder.

8. Once the urethral catheter is in situ, inflate the balloon of the urethral catheter with an appropriate amount of water for injection, if pre-filled syringes are not provided, as per manufacturer guidelines (usually 10 mL in two-way urethral catheters and up to 30 mL in three-way urethral catheters), and remove the soft-tipped hydrophilic guidewire from the urethral catheter and attach the urine collection bag. It is important to note that correct placement will be confirmed with urine noted at the end of the catheter. If urine is not seen at the distal end of the urinary catheter, the balloon must not be inflated, and incorrect placement of the urinary catheter must be assumed until proven otherwise and managed appropriately. Management of potentially incorrect urinary catheter placement may include removal of the catheter and reattempt or insertion via direct visualisation in the form of cystoscopy.

## Discussion

In most cases, the insertion of a urethral catheter is a relatively straightforward procedure, although this is further enhanced with the more experience an individual has gained. However, at the point the procedure is referred to the on-call doctor, there will have been multiple unsuccessful attempts already by clinicians of variable experience and skill. Hence, there is an increased risk of infection as well as damage to the urethra, including false passages, prior to the on-call doctor even having attempted the difficult urethral catheter. Therefore, it is vital that care is taken to minimise further unsuccessful attempts and prevent any further damage. As a result, any tips and tricks that will help an individual be both successful and safe without compromising patient safety are vital.

The insertion of a three-way urethral catheter is recommended in cases where haematuria with clots requires the patient to receive irrigation with or without bladder washouts. The method described above, which incorporates the use of a soft-tipped hydrophilic guidewire to help the passage of the catheter, can not only give reassurance that the urethral three-way catheter is in situ but can also lead to increased procedure success and improved confidence of the clinician performing the procedure.

Soft-tipped hydrophilic guidewire-assisted techniques, with or without the use of a dilator, will allow the user some confidence that the urethral catheter is in the bladder and that any potential damage to the urethra, including false passages, is minimised [9]. However, there can never be 100% confidence in any procedure and a cautious approach must always be taken.

Minimising patient harm is paramount to good healthcare. The use of a soft-tipped hydrophilic guidewire-assisted technique, when compared to multiple blind attempts, will lead to a reduced number of false passages, urethral trauma, and urethral stricture formation. These potential risk reductions will ultimately lead to reduced hospital stays and costs to the NHS [3,10].

The costs of different equipment and methods used for urethral catheterisation are shown in Table 2. All values are given in Great British Pound Sterling (£). The cost is based on tariff prices within a small district hospital in the NHS. Prices are true at the time of writing.

**Table 2:** Cost comparison of different urethral catheters used in the insertion of a urethral catheter when using a hydrophilic soft-tipped guidewire and urethral dilator. PFS = pre-filled syringe

Catheter type	Catheter costs (£)	18-G cannula (£)	Soft-tipped hydrophilic guidewire (£)	Urethral dilator 10–16 F (£)	Urethral dilator 16 F (£)	Total cost (£)	Soft-tipped hydrophilic guidewire + catheter (without urethral dilators) (£)
18-F Foley + PFS	04.81	00.56	36.00	47.80	47.80	136.97	41.37
20-F three-way catheter balloon	08.46	00.56	36.00	47.80	47.80	140.62	45.02
18-F two-way council Foley catheter	22.58	00.56	36.00	47.80	47.80	277.59	59.14
Tiemanns tipped catheter	01.61	00.56	36.00	47.80	47.80	133.77	38.17

Table 2 shows the cost comparison of different techniques of urethral catheterisation. The column labelled “Catheter costs” is the cost comparison of urethral catheterisation alone, without the use of soft-tipped hydrophilic guidewires or urethral dilators. The column labelled “Total cost” is the cost comparison of urethral catheterisation when using a hydrophilic soft-tipped guidewire and urethral dilators. The column labelled “Soft-tipped hydrophilic guidewire + catheter (without urethral dilators)” shows a cost comparison for urethral catheter insertion using soft-tipped hydrophilic guidewire alone without urethral dilators. Table 2 shows that there is a substantial increase in cost when using the soft-tipped hydrophilic guidewire technique, with or without dilators, when compared to other methods which do not utilise the hydrophilic soft-tipped guidewire approach. However, when compared to the cost of prolonged hospital admission due to injury caused by unsuccessful attempts, the use of a soft-tipped hydrophilic wire-guided technique in the particularly difficult catheter candidate appears to be cost-effective, while also increasing patient safety and reducing the risk of potential harm.

Council-tip and open-tip urethral catheters allow the bypassing of step 3 [2,11]. However, these are not always available to individuals, especially at smaller district general hospitals, as was the case in our situation. The (lack of) availability of council-tip/open-tip urethral catheters at some hospitals means the above approach of using a peripheral cannula to prepare the urethral catheter allows this method to be available to more clinicians in the ward, especially those in smaller hospitals. Consequently, this approach minimises the need to transfer patients to another hospital, thus improving patient care and reducing infection transmission and costs [12].

The district general hospital in which the above-mentioned method was employed utilised this approach to insert urethral catheters in 15 male inpatients in a six-month period of review (between August 2020 and February 2021), with 14 cases performed in a ward setting and one case performed in the accident and emergency department. In all cases, traditional methods of urethral catheterisation were unsuccessful. We found a 100% success rate in the insertion of the urethral catheter when the soft-tipped hydrophilic guidewire approach was employed. Of the 15 patients in whom this method was successful, 26% (n = 4) of the patients had a three-way catheter inserted successfully with the above-mentioned method using a soft-tipped hydrophilic guidewire approach. Follow-up of the 15 inpatients where this technique was utilised showed no adverse effects. A limitation of this data is that all attempts of insertion of a urethral catheter using a soft-tipped hydrophilic guidewire (n = 15) were done either by a urology consultant or under the supervision of a urology consultant.

Environmental benefits of the use of a guidewire include reduced numbers of catheters due to increased likelihood of success. This will reduce landfill/waste production, especially as catheters have little in the way of biodegradable properties [13].

## Conclusions

The use of a hydrophilic guidewire-assisted technique to insert a urinary catheter is both a safe and easy option for those who have had repeated unsuccessful attempts. The technique described above, if employed correctly, will allow for the safe urethral catheterisation of a patient without creating a strain on the already limited resources. It will also reduce the risk of catheter-associated complications and improve the overall experience of the patient.

The ease of access to equipment and the level of skill required for the procedure imply that it would be a good option for those who are asked for advice to provide support in difficult urethral catheterisation cases. In our experience, filiform and follower can be an alternative method for difficult urethral catheterisation; however, this method is not commonly performed in the NHS.

It is important to note that the above technique is not recommended in patients with polytrauma or where a urethral perforation/acute injury is suspected. Therefore, it should be used with caution in the emergency room setting. These patients should be managed by the appropriate team which would include a urologist and/or an interventional radiologist with the possibility of the patient requiring intervention in the form of a suprapubic catheter.
